# Tadalafil-Loaded Self-Nanoemulsifying Chewable Tablets for Improved Bioavailability: Design, In Vitro, and In Vivo Testing

**DOI:** 10.3390/pharmaceutics14091927

**Published:** 2022-09-12

**Authors:** Hany S. M. Ali, Sameh A. Ahmed, Abdulmalik A. Alqurshi, Ali M. Alalawi, Ahmed M. Shehata, Yaser M. Alahmadi

**Affiliations:** 1 Department of Pharmaceutics and Pharmaceutical Technology, College of Pharmacy, Taibah University, Al-Madinah Al-Munawarah P.O. Box 344, Saudi Arabia; 2 Department of Pharmaceutics, Faculty of Pharmacy, Assiut University, Assiut 71526, Egypt; 3 Department of Pharmacognosy and Pharmaceutical Chemistry, College of Pharmacy, Taibah University, Al-Madinah Al-Munawarah P.O. Box 344, Saudi Arabia; 4 Department of Pharmaceutical Analytical Chemistry, Faculty of Pharmacy, Assiut University, Assiut 71526, Egypt; 5 Department of Pharmacology and Toxicology, College of Pharmacy, Taibah University, Al-Madinah Al-Munawarah P.O. Box 344, Saudi Arabia; 6 Department of Pharmacology and Toxicology, Faculty of Pharmacy, Beni-Suef University, Beni-Suef 62521, Egypt; 7 Department of Clinical and Hospital Pharmacy, College of Pharmacy, Taibah University, Al-Madinah Al-Munawarah P.O. Box 344, Saudi Arabia

**Keywords:** tadalafil, self-nanoemulsifying, solidification, chewable tablets, bioavailability

## Abstract

This research aimed to develop innovative self-nanoemulsifying chewable tablets (SNECT) to increase oral bioavailability of tadalafil (TDL), a nearly insoluble phosphodiesterase-5 inhibitor. Cinnamon essential oil, PEG 40 hydrogenated castor oil (Cremophor^®^ RH 40), and polyethylene glycol 400 served as the oil, surfactant, and cosurfactant in the nanoemulsifying system, respectively. Primary liquid self-nanoemulsifying delivery systems (L-SNEDDS) were designed using phase diagrams and tested for dispersibility, droplet size, self-emulsifying capability, and thermodynamic stability. Adsorption on a carrier mix of silicon dioxide and microcrystalline cellulose was exploited to solidify the optimum L-SNEDDS formulation as self-nanoemulsifying granules (SNEG). Lack of crystalline TDL within the granules was verified by DSC and XRPD. SNEG were able to create a nanoemulsion instantaneously (165 nm), a little larger than the original nanoemulsion (159 nm). SNECT were fabricated by compressing SNEG with appropriate excipients. The obtained SNECT retained their quick dispersibility dissolving 84% of TDL within 30 min compared to only 18% dissolution from tablets of unprocessed TDL. A pharmacokinetic study in Sprague–Dawley rats showed a significant increase in C_max_ (2.3-fold) and AUC_0–24_ _h_ (5.33-fold) of SNECT relative to the unprocessed TDL-tablet (*p* < 0.05). The stability of TDL-SNECT was checked against dilutions with simulated GI fluids. In addition, accelerated stability tests were performed for three months at 40 ± 2 °C and 75% relative humidity. Results revealed the absence of obvious changes in size, PDI, or other tablet parameters before and after testing. In conclusion, current findings illustrated effectiveness of SNECT to enhance TDL dissolution and bioavailability in addition to facilitating dose administration.

## 1. Introduction

Erectile dysfunction (ED) is known as the consistent incapability to get and sustain erection as part of the broader process of male sexual function [1]. ED is a major global health concern that affects about 20–40% of men between the ages of 60 and 69 and more than 50% of men over the age of 70 [2]. According to a reported study, ED is strongly and independently linked to an elevated threat of coronary heart disorders, stroke, additional cardiovascular illnesses, and overall mortality [3]. Furthermore, ED affects quality of life substantially and is linked to depression, anxiety, and a loss of self-esteem, necessitating a proper therapy [4]. In addition, ED is a common complication among diabetic males. Another study showed that diabetic patients had an ED prevalence rate of 30–50%, which could be attributed to neuropathy and peripheral vascular dysfunction [5].

Tadalafil (TDL) is a phosphodiesterase-5 (PDE5) inhibitor that has been licensed for the treatment of erectile dysfunction [6]. TDL is considered the most potent and more selective compared to other PDE5 inhibitors [7]. TDL possesses a less inhibitory action for PDE6, with less than 0.1% incidence of vision side effects when compared to sildenafil and vardenafil [8]. As a result, TDL is clinically accepted to manage erectile disfunction even in problematic cases [9]. TDL is classified as a class II medication by the Biopharmaceutics Classification System (BCS), having poor solubility and high permeability characteristics [10]. Despite its good permeability, TDL bioavailability is limited by poor solubility and dissolution rate, resulting in erratic absorption, fluctuation of drug blood levels, and unreproducible effect clinical outcomes [11].

Several techniques have been developed and tested to improve dissolution of poorly soluble drugs. Out of these techniques, self-nanoemulsifying drug delivery systems (SNEDDS) have acquired a wide popularity in pharmaceutical research [12,13,14,15,16,17]. SNEDDS can be defined as isotropic mixes of drug, lipid, and surfactants, often containing one or more hydrophilic co-solvents or co-emulsifiers. When gently agitated in aqueous environments, such systems are able to generate ultrafine oil droplets containing the active ingredients [18]. Due to the minimal free energy needed for this process, self-nano emulsification will happen spontaneously [19]. The enhanced interfacial area of medicated nano-sized oil droplets accelerates drug dissolution and consequently improves its bioavailability. The presence of the lipid and surfactant also enhances permeability through biological membranes [20]. When orally administered, agitation achieved by digestive motility of stomach and intestines is sufficient for in vivo emulsification [21]. Nonetheless, there are some drawbacks challenging the use of conventional liquid SNEDDS (L-SNEDDS). During storage, the drug or components’ precipitation, potential interactions between the filling and the capsule shell, and formulation instability may occur [13]. The main strategy applied to overcome these challenges is to transform L-SNEDDSs into solid dosage SNEDDS formulations (S-SNEDDS). Such a transformation combines the benefits of SNEDDS (such as improved solubility and bioavailability) with those of solid dosage forms, i.e., helps reduce production cost, improve formulation stability, simplify manufacturing, allow accurate dosing, and improve patient compliance [22,23]. S-SNEDDS employ various solidification processes, such as adsorption on carriers, [24,25], spray drying [26], melt granulation [27,28,29], and andextrusion–spheronization [30] to integrate liquid or semisolid components into powders. The generated solid SNEDDS can further be formulated into free-flowing powders, granules, pellets, tablets, solid dispersions, microspheres, and nanoparticles [31,32]. Yet, physical adsorption remains the simplest technique for S-SNEDDS formulation. The coupling of S-SNEDDs strategy as well as their formulation as patient-friendly chewable tablets is valuable for promoting drug disintegration and dissolution, hence increasing bioavailability.

Some TDL-SNEDDS formulations have already been described in the literature to enhance solubility and dissolution of the drug [10,33]. However, the impact of SNEDDS formulations on drug bioavailability has not been studied. The rationale of the current work was to develop chewable tablets based on a self-nanoemulsifying technology to accomplish quick drug disintegration and make tadalafil administration more convenient through using chewable and palatable tablets. A thorough review of the literature and patent databases did not reveal research regarding TDL bioavailability enhancement using SNEDDS-based chewable tablets. Accordingly, the aim of the present study was to manufacture a novel self-nanoemulsifying chewable tablet (TDL-SNECT) to facilitate administration and improve TDL dissolution, stability, and bioavailability simultaneously. Here, TDL was firstly incorporated within a L-SNEDDS, which was then solidified into self-nanoemulsifying granules (SNEG) using a combination of silicon dioxide/microcrystalline cellulose carrier before being compressed into a self-nanoemulsifying chewable tablet (TDL-SNECT). Various in vitro and in vivo characterization tests were performed to discover TDL release and absorption profiles.

## 2. Methodology

### 2.1. Materials

Tadalafil (98%) was kindly gifted by SAJA Pharmaceuticals, (Jeddah, Saudi Arabia). Cinnamon essential oil, orange oil, lemon oil, and peppermint oil were brought from Now foods (Bloomingdale, IL, USA). Propylene glycol dicaprylate/dicaprate (Labrafac^TM^ PG), caprylocaproyl macrogol-8-glyceride (Labrasol^®^), propylene glycol mono caprylate (capryol^®^ 90) were obtained from UFC Biotechnology (Amherst, NY, USA). Oleic acid was obtained from Merk KGaA (Darmastadt, Germany). Polyoxy 40 hydrogenated castor oil (Cremophor^®^ RH 40), polyethylene glycol 200 (PEG 200) and polyethylene glycol 400 (PEG 400), polyoxyethylene sorbitan monolaurate (Tween^®^ 20), polyoxyethylene sorbitan monopalmitate (Tween^®^ 40) and polyoxytheylene-20-sorbitan monooleate (Tween^®^ 80), and mannitol and magnesium stearate were bought from Sigma-Aldrich Chemie-GmbH (Steinheim, Germany). Silicon dioxide was bought from spectrum chemical MGF Corp (New Brunswick, NJ, USA). Microcrystalline cellulose (MCC PH-101) was purchased from FMC, (Cork, Ireland). Crospovidone (Polyplasdone^TM^ XL10) was obtained from Ashland Specialty Ingredients (Wilmington, DE, USA). Steviana powder was procured from (Bawazir Factory Jeddah, Jeddah, Saudi Arabia). Sorbitan monooleate (Span^®^ 80) was obtained from Loba Chemie (Mumbai, India). The internal standard, sildenafil (SLD, 97.5%), and pea nut oil were purchased from Acros Organics (Geel, Belgium). Ammonium acetate and formic acid, propylene glycol, and glycerin were obtained from Sigma Aldrich (Seelze, Germany). Ethyl acetonitrile (and HPLC grades) was purchased from Cromasolv (Honeywell, MI, USA). A Millipore water filtration system (Bedford, MA, USA) was used to obtain ultra-pure water. Other chemicals and materials were of pharmaceutical grades and were utilized in their original forms.

### 2.2. TDL Quantification

For in vitro samples, a validated high-performance liquid chromatography (HPLC) assay was applied for TDL quantification [34]. The HPLC system consisted of PU 2080 pump (Jasco Corporation, Tokyo, Japan) with UV/VIS detector (UV 2075 plus, Jasco Corporation, Tokyo, Japan) in the range of 200–400 nm. A reverse-phase C18 column (250 × 4.6 mm, 5 μm) was utilized for quantification (HiQ Sil, Kya Tech Corporation, Tokyo, Japan). The mobile phase for TDL separation was made up of phosphate buffer pH 3.2 and acetonitrile (50:50 *v*/*v*). The mobile phase flow rate was kept constant at 1.0 mL/min, and the detector was set at 295 nm. This method was validated in terms of linearity, accuracy, precision, and specificity using criteria from the International Conference on Harmonisation. For in vivo samples, TDL was quantified according to a reliable, accurate, and precise ultraperformance liquid chromatography method with electrospray ionization and tandem mass detection (UPLC-MS/MS) method [35]. Briefly, for chromatographic separation, an Agilent Eclipse Plus C18 RRHD (50 mm × 2.1 mm, 1.8 m) column (Agilent Technologies, Santa Clara, CA, USA) was used with isocratic elution using 2.0 mM ammonium acetate and acetonitrile (55:45, *v*/*v*) with 0.1% formic acid at a flow rate of 0.7 mL/min. Multiple reaction monitoring at transitions of m/z 390.4 268.3 for TDL and m/z 475.3 283.3 for sildenafil (SLD, internal standard). The linearity, accuracy, precision, and specificity of this approach were completely verified throughout a concentration range of 5–1000 ng mL^−1^. The Mass Hunter Quantitative Data Analysis program was used to process the data (Agilent Technologies, Santa Clara, CA, USA).

### 2.3. Solubility Studies

Solubility of TDL was determined in distilled water and in different SNEDDS components, oils, surfactants, and co-surfactants. An excess quantity of TDL was added to 2 g of each vehicle and shaken for 48 h at room temperature (25 ± 0.5 °C) in screw-cap glass vials using an Ika^®^ KS 260 B (Staufen, Germany) shaker at a mixing rate of 100 rpm. Each system was then centrifuged at 10,000 rpm for a period of 15 min (Centrifuge Z 206 A; Hermle Labortechnik GmbH, Wehingen, Germany). The supernatant was filtered using a 0.45 µm syringe filter, and an aliquot of 0.1 mL was collected and diluted by methanol. Quantification of TDL was performed as previously described (Section 2.2). For each vehicle, findings were reported as mean values and standard deviation (S.D.) of three samples.

### 2.4. Screening of Surfactants and Cosurfactants

Surfactants and cosurfactants with reasonable TDL solubilizing actions were tested for their capacity to emulsify the oil phase. The percentage transparency and ease of emulsification were used to determine surfactant emulsification efficiency. Briefly, equal amounts of the tested surfactants were combined with fixed amounts (300 mg) of the oil. The mixes were gently heated at 45 °C to homogenize the components. Then, 50 mg of each combination was diluted to 50 mL in a stoppered conical flask with deionized water. Ease of emulsification was determined by the number of flask inversions necessary to form a homogenous nanoemulsion. The percentage transparency of the formed mixes was measured spectrophotometrically (UV6100 PC, EMC lab, Duisburg, Germany) at 650 nm using deionized water as a blank. The resulting mixtures were then visually examined for turbidity and phase separation. Following selection of the oil and surfactant, similar procedures were applied to screen different cosurfactants to judge their co-emulsification capacity. Combinations of 50 mg cosurfactant, 100 mg surfactant, and 200 mg oil were created and assessed as formerly explained.

### 2.5. Construction of Phase Diagrams

The oil, surfactant, and cosurfactant identified from prior screening were utilized to generate the pseudo-ternary phase diagrams at room temperature using the water titration technique. S_mix_ components, surfactant, and co-surfactant, were weighed in glass vials at different Km (S/Cos) ratios, i.e., 0.5, 1, 2, and 4, and stirred for 15 min at 45 °C with a magnetic stirrer to allow full melting of Cremophor^®^ RH40 (CR, Sigma-Aldrich Chemie-GmbH, Steinheim, Germany). The oil phase and each of the S_mix_ ratios were fully mixed in various weight fractions (from 1:9 to 9:1) and then slowly titrated against the aqueous phase. During titration, samples were stirred to allow equilibration and then inspected visually for transparency. Systems were identified as nanoemulsions if their average droplets sizes were equal to or less than 200 nm and generated clear (% transmittance ≥ 95%) or were translucent (% transmittance ≥ 90%) dispersions [36]. Phase diagrams of the self-emulsifying region were constructed by ProSim Ternary Diagram software (ProSim, Toulouse, France).

### 2.6. Preparation and Characterization of TDL L-SNEDDS

Following identification of the self-emulsifying area, the optimal L-SNEDDS component ratios were chosen for drug loading, and subsequent optimization. S_mix_ combinations were gently heated at 45 °C to homogenize components and then added to the oil containing calculated amounts of TDL and mixed by a vortex mixer (VSM-3, Pro Scientific Inc, Oxford, CT, USA). The produced TDL-SNEDDS were stored in a firmly sealed container at 25 °C. Further research was conducted on stable formulations. Composition of L-SNEDS formulations is presented in Table 1.

#### 2.6.1. Dispersibility and Transmittance

A dispersibility test was conducted to evaluate TDL L-SNEDDS formulation (F1–F6) capability of self-emulsification. A dropping pipette was used to introduce 1 g of each formulation dropwise to a 500 mL, gently agitated (50 rpm) aqueous medium at 37.0 ± 0.5 °C. The time necessary for the L-SNEDDS to fade away was recorded [37]. Nanoemulsification abilities were visually classified as the following: grade A: for clear or slightly blue emulsions; grade B: for slightly less clear emulsions with a bluish-white shade; grade C: for brilliant white emulsions similar to milk; grade D: for greyish-white emulsions having a faintly greasy look; grade E: for dispersions of limited emulsification ability with big oil droplets at their surfaces [38]. Percentage transmittance was spectrophotometrically assessed (UV6100 PC, EMC lab, Duisburg, Germany) after a 100-times dilution using double distilled water at 638 nm [39].

#### 2.6.2. Robustness to Dilution

The influence of dilution on nanoemulsion properties was investigated for F1-F6 to imitate in vivo dilution behavior. Distilled water, 0.1 N hydrochloric acid, and phosphate buffer pH 6.8 were used to dilute 1 g of each tested formulation at dilution levels of 10, 100, and 1000. The diluted solutions were mixed using a magnetic stirrer (100 rpm) at 37 °C to obtain perfect homogeneity and to simulate body temperature. The produced dispersions were kept at room temperature (25 ± 0.5 °C) for 24 h before being visually inspected for evidence of phase separation [40,41].

#### 2.6.3. Droplet Size, Polydispersity Index, and Zeta Potential

Droplet size, PDI, and zeta potential of F1-F6 were determined by Microtrac S3500 (Microtrac Inc., Montgomeryville, PA, USA). Following dilution with double-distilled water (1:100, *v/v*), to avoid the effect of multiple scattering, nanoemulsions were measured three times for 120 s at 25 °C [42,43].

#### 2.6.4. Thermodynamic Stability Studies

Centrifugation tests (5000 rpm for 30 min), heating–cooling cycles (45 °C and at 0 °C for 48 h), and freeze–thaw cycles (−21 °C and 25 °C for 24 h) were used to conduct thermodynamic stability investigations [44]. The absence of phase separation implies that the L- SNEDDS formulation is stable.

### 2.7. TDL-Loaded Self-Nanoemulsifying Granules (TDL-SNEG)

Based on previous evaluation tests, an optimized TDL-SNEDDS formulation was considered for further processing into solidified dosage forms. The selected formula was transformed into free-flowing granules using an optimized combination of mixed carriers, (silicon dioxide and MCC). Different carrier ratios were tested in preliminary experiments, and the ratio 1:1 (*w*/*w*) was found the best to obtain free-flowing granules. In a glass mortar, the solid mixture was wetted by gradual addition of the L-SNEDDS to obtain a homogeneous damp mass. The mass was subsequently passed through a sieve (mesh number 22) to obtain uniform free-flowing self-nanoemulsifying granules (TDL-SNEG). The obtained granules were dried to constant weight at 40 °C in an oven (Binder oven, Tuttlingen, Germany) for 24 h to reduce the moisture level to less than 2% (Mettler Toledo HB43-S, Greifensee, Switzerland). 

#### 2.7.1. Reconstitution Potential

A sample (100 mg) from the prepared TDL-loaded SNEG was allowed to disperse in 10 mL of distilled water under gentle stirring. A 1 mL aliquot was taken and placed in a sample cell to quantify droplet size and PDI. Results were compared with those of the optimized batch of TDL L-SNEDDS.

#### 2.7.2. Micromeritic Properties 

##### Angle of Repose

SNEG were deposited in a funnel with a burette stand and let to run freely over graph paper, making a heap. The heap’s height (*H*) and radius (*R*) were measured. The angle of repose was computed by applying Equation (1):

(1) $$\theta =tan^{-1}\;\frac{H}{R}$$

The bulk $\left(\rho_{b}\right)$ and tapped densities $\left(\rho_{tap}\right)$ were used to determine Carr’s index (CI) and Hausner ratio (HR) values. Using a tapped density device, the fixed weight of each sample was tapped into a 50 mL measuring glass cylinder. The following Equations (2) and (3) were used to compute CI and HR values.

(2) $$CI=\left[\frac{\rho_{tap}-\rho_{b}}{\rho_{tap}}\right]X100$$

(3) $$CHR=\frac{\rho_{tap}}{\rho_{b}}$$

#### 2.7.3. Solid State Characterization

DSC thermograms of unprocessed TDL, silicon dioxide, MCC, their physical mixture, and TDL-SNEG were achieved employing a Netsch DSC apparatus (Netsch F3 Maia^®^, Selb, Germany). Samples in pin-holed aluminum hermetic pans and reference pans were heated in the heating chamber from 0 °C to 400 °C at a 10 °C/min heating rate. Crystalline state characteristics were also confirmed by a Shimadzu XRD 6000 diffractometer (Shimadzu Corporation, Kyoto, Japan). Analysis of powders was performed using a copper anode operated at a voltage of 40 kV with a current of 30 mA radiation. X-ray powder diffractograms (XRPD) were generated in the 2 θ angle range of 10–60° using a speed of 0.04/min. XRDP analysis was repeated for TDL-SNEG sample after storage for 3 months at 40 ± 2 °C and 75% relative humidity. SNEGS morphology was evaluated using a field emission scanning electron microscope (FESEM, LEO 1530170, Carl Zeiss SMT Inc., Oberkochen, Germany). To increase conductivity during FESEM processing, samples of the resulting solidified powders were sputter coated with carbon. Micrographic pictures were captured at various magnifications using a 5 kV acceleration voltage. For transmission electron microscope (TEM) imaging, a sample from reconstituted SNEGs was put over the formvar-coated grid and negatively stained with a 2 percent aqueous solution of phosphotungstic acid and allowed to dry for 5 min at room temperature before TEM examination (JEOL JEM-HR-2100, JEOL, Ltd., Tokyo, Japan).

### 2.8. Tablet Formulations 

**Figure 1:**
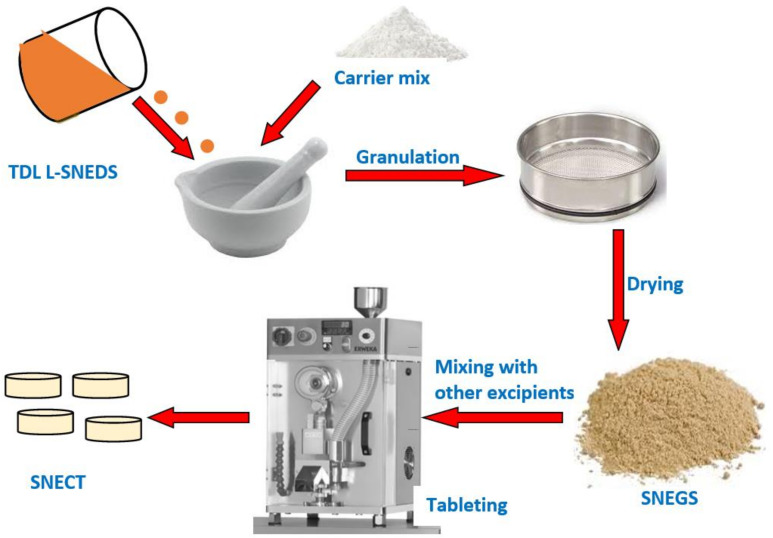
Preparation steps of tadalafil-loaded self-nanoemulsifying chewable tablets (SNECT).

To prepare SNECT formulation, SNEG were mixed gently using a mortar and a pestle with other tablet ingredients and compressed at 10 kilonewton (kN) via a single punch tableting instrument (Erweka GmbH D-63150 Heusenstamm Germany). Inactive ingredients include mannitol (a diluent for chewable tablets), Polyplasdone^TM^ XL (an extra granular disintegrant), steviana powder (a low-caloric sweetener), and magnesium stearate (as a lubricant). To investigate the impact of nanoemulsification on TDL dissolution, directly compressed tablets (DCT) were manufactured identically but not including the self-emulsifying system. A schematic diagram of solidification and tableting procedures is displayed in Figure 1, while formulations details are presented in Table 2. 

#### 2.8.1. Tablets Characterization

Tablet hardness test was conducted using a hardness tester (Erweka GmbH, Heusentamm, Germany), and tablet friability was assessed by a Pharma Test friabilator (Pharma Test, Hainburg, Germany), with a total of 300 rotations [44]. Disintegration of SNECT was analyzed in distilled water at 37 °C using PTZ-S single-basket tablet disintegration tester (Pharma Test, Hainburg, Germany). Tablets were monitored until they fully disintegrated [44].

#### 2.8.2. Dissolution Study

TDL dissolution from manufactured SNECT was compared to L-SNEDDS, DCT, and a commercial product (Cialis^TM^ 2.5 mg, Lilly, LLC, Indianapolis, IN, USA) in 500 mL of 0.1 N HCl buffer dissolution media using a USP Dissolution Tester at 37 °C with a paddle speed of 100 rpm [45,46]. At 5, 10, 15, 30, 45, 60, 90, and 120 min, samples (5 mL) were removed and replaced with equal volumes of fresh medium. To separate undissolved excipients, samples were centrifuged at 6000 rpm for 15 min. The supernatant was filtered through a 0.2 µm membrane filter (Millipore, Mumbai, India), diluted appropriately, and then analyzed using the HPLC procedures explained in Section 2.2. 

#### 2.8.3. Stability Tests

##### Stability in Simulated GI Fluids

The stability of TDL-SNECT was checked against simulated GI fluids (i.e., simulated gastric fluids (SGF); pH 1.2 and simulated intestinal fluids (SIF); pH 6.8). This test was performed by taking 100 mg of the formulation and subjecting to dilution by 25 mL of the mentioned fluids. The mixtures were equilibrated for 2 h and 6 h in SGF and SIF, respectively. After equilibration, the samples were diluted 10 times with tested fluids and analyzed for ADS and PDI [47].

##### Dilution Stability in Simulated GI Fluids

TDL-SNECT formulation was assessed for stability against dilution in SGF and SIF to different folds. First, 100 mg of prepared formulations were reconstituted in 10 mL of SGF or SIF and then subjected to 200-, 400-, 600-, and 800-fold dilutions with the corresponding fluids and kept aside for 2 h to attain equilibrium and considered for analysis of average droplet size (ADS) and PDI [47].

##### Accelerated Stability Tests

The developed TDL-SNECT was subjected to accelerated stability for three months, in accordance with the ICH guidelines. TDL-SNECT were kept in sealed glass vials and held in a stability chamber maintained at 40 ± 2 °C and 75% relative humidity (RH). At the end of three months, SNECT were assessed for appearance, tablet characterization test, ADS, and PDI [48].

#### 2.8.4. Oral Bioavailability Study

##### Animals and Samples Collection

Ethics Committee at College of Pharmacy, Taibah University approved the in vivo study procedures prior starting the investigation (Approval number: COPTU-REC-17-20210705). In vivo study was carried out in the animal house at College of Pharmacy, Taibah University in accordance with the criteria for the care and use of laboratory animals contained in the National Academies Press Guide for the care and use of laboratory animals, 8th edition, Washington, DC, USA. Experimental animals were distributed into 3 groups, each group containing 5 male Sprague–Dawley rats (250 ± 20 g). Before conducting tests, rats were housed in the animal house for 5 days to allow them to acclimate. Rats were fed a conventional rat diet and had free access to tap water while being kept in a controlled environment (22 °C ± 3 °C, 50% ± 5% relative humidity, and a 12 h light/dark cycle). Prior to the trial, the rats were fasted for an overnight period. Tablets were dispersed in appropriate volumes of deionized water and administered to rats (1 mg of TDL per kg of body weight [49]) via oral gavage with blunt intragastric tubing to ensure correct dose administration; this was followed by 0.5 mL of deionized water for cleaning reasons. The commercial TDL formulation was given to group I of Sprague–Dawley rats, while groups II and III received TDL–SNECT and TDL–DCT, respectively. Blood samples were obtained in heparinized Eppendorf tubes at intervals of 0.5, 1, 2, 4, 8, 12, and 24 h. The samples were centrifuged for 10 min at 15,000 rpm and stored at −20 °C until analyzed.

##### Processing and Quantification

A 100 μL aliquot of plasma was placed into a 1.5 mL Eppendorf tube, followed by 10 μL of the internal standard (IS) solution (SLD; 500 ng/mL) and 200 μL of methanol. The contents of the Eppendorf tube were vortex mixed for 20 s before being centrifuged at 15,000 rpm for 5 min at room temperature (25 ± 0.5 °C). TDL was quantified according to a reliable, accurate, and precise UPLC-MS/MS method as explained in Section 2.2 [35]. 

##### Pharmacokinetic Analysis

The pharmacokinetic parameters, C_max_ (the maximum peak of plasma drug concentration), and the time point of peak concentration (t_max_) were read from plasma concentration data. The trapezoidal rule was used to compute the area under the plasma concentration–time curve in the first 24 h (AUC_0–24 h_). SPSS 16 Statistics software was used to run a one-way ANOVA (SPSS Inc., Chicago, IL, USA). A statistically significant *p*-value of 0.05 was used.

## 3. Results and Discussion

### 3.1. Solubility Study

**Figure 2:**
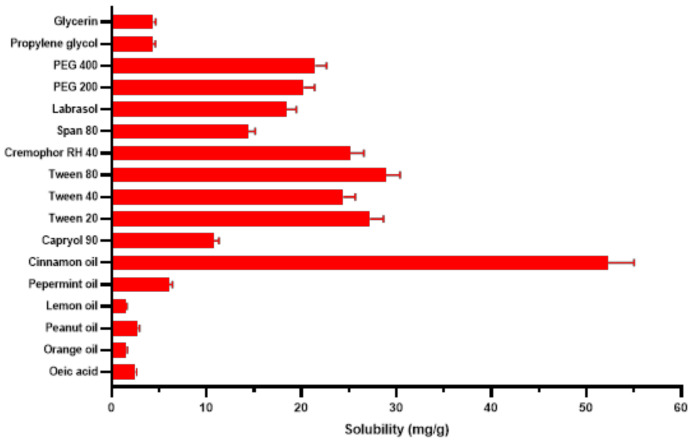
Tadalafil solubility in different oils, surfactant, and co-surfactants.

The capability to dissolve considerable drug amounts is crucial to select the oil component of nanoemulsions, particularly for orally administered ones. If the surfactant and cosurfactant noticeably contribute to drug solubilization, dilution in GIT could lower their solvent ability and precipitate the drug. The aqueous solubility of TDL was found to be 0.01 ± 0.009 mg·g^−1^. Results of solubility study are graphically displayed in Figure 2. Amongst tested essential oils, the highest solubility of TDL was observed in cinnamon essential oil, i.e., CEO (52.3 ± 2.7 mg·g^−1^), while its least solubility was in lemon oil (1.49 ± 0.11 mg·g^−1^). CEO was also successfully selected as the lipid component of SNEDDS due to its solubilizing potential for a number of drugs such as rosuvastatin calcium [50] and candesartan cilexetil [51]. Higher oil solubility of poorly water-soluble drugs helps stabilize formulations with efficient dose optimization and cost-effective delivery [52]. A lesser amount of CEO will be required to solubilize TDL doses with the reduction of any possible unwanted actions of the essential oil. Besides good solvent capacity, CEO and its major compound, cinnamaldehyde, were found effective to induce relaxation in human and rat corpus cavernosum, improving erectile functions [53]. The chosen surfactant should decrease the interfacial tension to assist the dispersion process throughout nanoemulsion formulation. Owing to their relatively low toxicity, a variety of hydrophilic nonionic surfactants with a relatively high HLB, such as labrasol^TM^, polysorbates (Tween^®^), and polyoxyls (Cremophor^®^), have been extensively used in SNEDDS formulations [13]. In this study, Tween^®^ 80, Tween^®^ 20, Cremophor^®^ RH 40 (CR), Span^®^ 80, and labrasol were selected for emulsification study, as they showed good solubility potential for TDL. Findings of the % transmittance and the number of flask inversions (ease of emulsification) revealed that CR showed the highest emulsification ability towards (Table 3). The addition of appropriate cosurfactant improved spontaneity of emulsification via reducing the interfacial tension, fluidizing the hydrocarbon region of interfacial film, and decreasing the bending stress of interface [54]. Besides its good solubility potential for TDL, PEG 400 showed highest emulsification ability toward CEO (Table 3). Based on the previous findings, a system composed of CEO, CR, and PEG 400 as the oil, surfactant, and cosurfactant, respectively, was selected for further studies.

### 3.2. Phase Diagram

**Figure 3:**
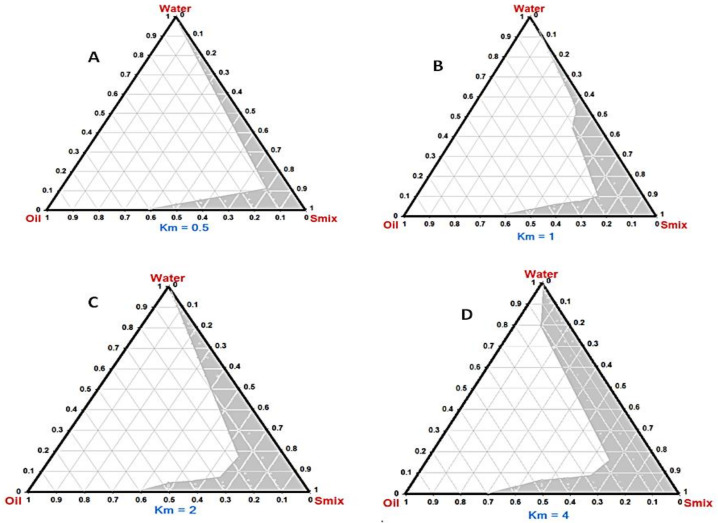
Ternary phase diagrams depicting the nanoemulsion region (NE, shadow areas). Km: Surfactant/cosurfactant mass ratio. (**A**) Km = 0.5:1, (**B**) Km = 1:1, (**C**) Km = 2:1, (**D**) Km = 4:1.

Phase diagrams were constructed to demarcate the regions of nanoemulsion and to determine the plausible ratios of surfactant/cosurfactant for the generating of a stable SNEDDS. The phase diagram is comprised of 100% of oil, S_mix_, and water in each corner (Figure 3). A larger shaded region signifies superior nanoemulsifying capability. Components were converted to mass percent (w%) prior to creating phase diagrams. Generally, nanoemulsification was found to be promising at a low level of the oil. The addition of surfactant and cosurfactant enhances the efficiency of emulsification owing to their higher hydrophilicity characters. SNEDDS prepared with Km = 4 (Figure 3D) showed more pronounced self-nanoemulsifying capability relative to other Km values, suggesting optimum emulsification gained by this ratio. At the proper Km ratio, packing of surfactant and cosurfactant at the o/w interface will be effective to reduce the interfacial tension and enhance fluidity of the interface [55].

### 3.3. Tadalafil-Loaded Liquid Self-Nanoemulsifying Delivery Systems (TDL L-SNEDDS)

Results of characterization of the prepared L- SNEDDS formulations (F1–F5) are displayed in Table 4. The emulsification rate is crucial for determining the system’s spontaneity of emulsification. When exposed to water dilution and modest GI tract motion, the SNEDDS should dissolve entirely and promptly [56]. Self-emulsification tests revealed that all L-SNEDDS formulations generate fine, bluish-white emulsions in short times (t < 1 min). Transparency is a prime requirement of SNEDDS preparations and necessitates a particular attention [57]. Percentage transparency (PT) values of the tested formulations were over 90%, with direct proportional to S_mix_ concentrations, which advocate the transparency of the system with fine particle size of nanoemulsion [58]. Except for F1, there was no evidence of precipitation or phase separation after diluting formulations with water, 0.1 N HCl, or phosphate buffer. Such findings encouraged appropriateness of the developed nanoemulsion for oral administration and subsequent GI fluids [59]. Moreover, this finding confirms that only CEO is responsible for TDL solubilization, and neither surfactant nor cosurfactant contribute in drug solubilization since dilution by GIT fluids diminishes the solvent capacity of the surfactant or cosurfactant [36]. Average droplet size (ADS) is an important measure of a nanoemulsion’s physical stability, as tiny droplets suggest a non-flocculating system. It also impacts the rate and degree of drug release and is a significant determinant in self-emulsification performance. The polydispersity index (PDI) represents the homogeneity of size distribution. Size analysis revealed that emulsion droplets are in nanometric range (between 32 ± 4.6 and 198 ± 8.7 nm), with a noticeable decrease with increasing S_mix_ concentration. Similarly, PDI values were larger for F1 and F2 and then decreased to smaller values other formulations. Higher values of particle size and PDI for F1 and F2 are attributed to the decreased S_mix_ efficiency to reduce the interfacial tension satisfactorily and generate uniformly sized nanoemulsions. The ADS in F5 and F6 is remarkably low. In this case, higher concentrations of S_mix_ are responsible for the formation of swollen micelle systems or microemulsions, in which the dispersed phase is integrated inside the core of surfactant micelles [60,61], which could affect the bioavailability of the drug subsequently. The measured values of ZP range from −4.7 ± 0.3 to −19.7 ± 0.41 mV. The reduced values of ZP is a plausible cause of the reduced stability of F1 and F2. Generally, under the different specified stress conditions, thermodynamic investigations indicated a good stability of the tested SNEDDS formulations (except F1 and F2), with no evidence of phase separation or precipitation (Table 4). 

### 3.4. Tadalafil-Loaded Self-Nanoemulsifying Granules (TDL-SNEG)

The usual preference in oral delivery of nanoemulsions is to select formulations with the lowest surfactant concentration. Thus, taking into account the aforementioned findings, it can be concluded that formulation F3, with oil:S_mix_ 1:2, demonstrates more prominent self-nanoemulsifying features with ADS, PDI, and ZP values of 174.2 ± 1.1 nm, PDI 0.246 ± 0.02, and −9.92 ± 1.2 mV, respectively. Hence, F3 was considered as the best self-emulsifying system to fabricate TDL-SNEG. Preliminary studies were carried out to identify the excipients suitable to develop SNEG. Because of its high porosity, small particle size, and large adsorptive capacity, silicon dioxide was frequently used to adsorb and convert L-SNEDDS into solid powder. However, silicon dioxide may affect the desorption process due to considerable interaction with the adsorbed SNEDDS [62]. MCC has good compatibility characteristics, providing tablets cohesion and strength. However, when used alone, MCC did not show a sufficient adsorption capacity toward L-SNEDDS. When mixed, silicon dioxide can form a dry, flowable powder by covering the wet MCC particles saturated with liquid [14]. Our trials concluded that the carrier mix silicon dioxide:MCC in 1:1 ratio showed a reasonable adsorption capacity and produced satisfactory SNEG. When diluted with water, SNEG retained the L-SNEDDS’s self-emulsification capability, producing a 165 ±3.7 nm sized nanoemulsion with a PDI value of 0.49 ± 0.1, which was slightly higher than the initial droplet size and PDI values of L-SNEDDS, at 159 ± 4.7 nm and 0.24 ± 0.03, respectively. Such a marginal rise in droplet size and PDI value assumes that the solidification of L-SNEDDS by adsorption on the carrier mix had no effect on its own emulsification ability. The angle of repose of SNEG was 28.3 ± 1.6, Carr’s index was 12.2 ± 1.1, and Hausner’s ratio was 1.23 ± 0.14 percent, indicating a good flow quality of the granules (Table 5).

**Figure 4:**
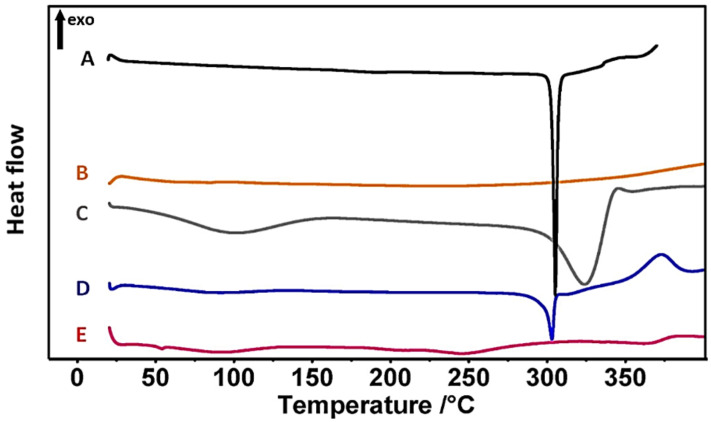
DSC thermograms of tadalafil (**A**), silicon dioxide (**B**), MCC (**C**), physical mixture (**D**), and tadalafil-loaded self-nanoemulsifying granules, SNEG (**E**).

Thermal properties of the TDL-SNEGS, unprocessed TDL, carriers, and the physical mixture were studied using DSC. TDL presented a sharp endothermic peak at 304 °C (Figure 4A), confirming the crystalline nature of the drug [63]. Silicon dioxide did not generate any endothermic peaks, suggesting its amorphous nature [62] (Figure 4B). MCC showed two endothermal peaks (Figure 4C). The first shallow, broad peak in the scanned region between 60 °C and 145 °C indicates desorption of water from the cellulose materials, while the second peak (325 °C) corresponds to melting or thermal decomposition of MCC [64]. The physical mixture showed a similar peak corresponding to TDL (Figure 4D). Nevertheless, no distinctive TDL peak was identified in solid SNEG thermogram at the studied temperature (Figure 4E), implying drug amorphization.

**Figure 5:**
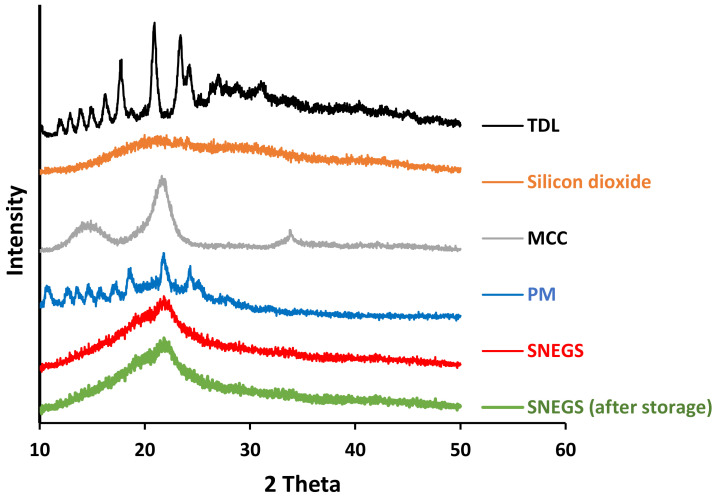
XRPD of tadalafil, silicon dioxide, MCC, physical mixture, and tadalafil-loaded self-nanoemulsifying granules (SNEGs).

The TDL crystalline state was additionally investigated by XRPD (Figure 5). The diffractogram of unprocessed TDL revealed sharp peaks at different (2θ) diffraction angles, i.e., 10.18°, 12.6°, 15.07°,16.25, 17.78°, 21.05°, and 24.25° [63], resulting in a typical crystalline pattern, whereas the pattern of silicon dioxide had no defined features that suggest a crystalline module. MCC revealed a pattern characteristics of cellulose, with diffraction peaks appearing at 2θ angles of 15.5 and 22 [64]. The physical mixture showed major characteristic crystalline peaks as TDL, with a reduced intensity due to reduced concentration of TDL in the mixture. However, crystalline peaks of TDL were absent in the self-nanoemulsifying granules (SNEG) diffractogram, indicating the presence of the drug in a dissolved and molecular dispersion within the SNEGs formulation. Overall data of DSC and XRPD confirmed the absence of TDL crystallinity within SNEG preparation. Adsorption of L-SNEDDS on the solid carriers converted TDL molecules from crystalline to amorphous form. The transition to an amorphous form is certainly a plausible reason for better TDL dissolution and biological availability [63]. Diffractogram of TDL-SNEG stored for 3 months at 40 ± 2 °C and 75% RH was identical to that of freshly prepared SNEG, indicating the absence of phase transition of TDL molecules to crystalline forms. This could be attributed to the high value of glass transition, Tg, of TDL (143.8 °C) [65].

**Figure 6:**
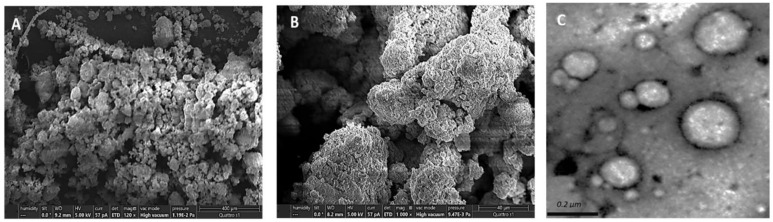
Scanning electron micrographs of tadalafil-loaded SNEGS: (**A**) ×120 and (**B**) ×1000; TEM image of reconstituted SNEG (**C**).

SNEG morphology is depicted in Figure 6. SNEG showed smooth granular particles with aggregated spherical particles and deep crevices. This demonstrated that the L-SNEDDS was absorbed within the carrier mix’s pores. Moreover, no distinguishable crystals were evident on solid SNEGS, implying that crystalline TDL has been transformed into amorphous form (Figure 6A,B). TEM analysis of the reconstituted SNEG revealed nanospherical droplets (Figure 6C). The observed droplets sizes in TEM image (Figure 6C) appeared smaller than the sizes obtained by dynamic light scattering (DLS) technique after reconstitution (Table 5). The discrepancy in the observed droplets size between the two measurement methods was predicted because DLS sizes include hydration layers, shells of the used polymers, or other stabilizers, resulting in increased overall droplet sizes [66].

### 3.5. Tadalafil-Loaded Self-Nanoemulsifying Chewable Tablets (TDL-SNECT)

#### 3.5.1. Characterization and Reconstitution Potential

**Figure 7:**
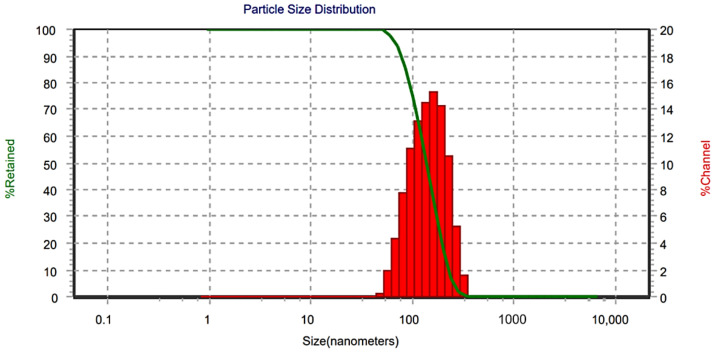
Size distribution after redispersion of tadalafil self-nanoemulsifying chewable tablets (TDL-SNECT) formulation.

The hardness and friability of the tablets were 4.23 ± 0.33 kg/cm^2^ and 0.29 ± 0.01%, respectively. The mean weight of SNECT was (700 ± 13.3 mg, *n* = 10), and no tablet showed a percentage deviation from the mean weight greater than 5%. The drug content was (96.42 ± 1.1) and was within the acceptable range of 95–105%. TDL-SNECT had a mean disintegration time of 49 ± 3.7 s. When subjected to an aqueous environment under mild agitation, SNECT formulation should rapidly and entirely disperse TDL-loaded droplets. The result of reconstitution potential of TDL-SNECT is shown in Figure 7. According to result, no marked variation was noticed in the size of generated droplets from SNECTs (169 ± 8.7) relative to those obtained from L-SNEDDs (159 ± 4.7 nm) or SNEGS (165 ± 3.7 nm). The calculated PDI was slightly higher for the redispersed tablets (0.53 ± 0.05). The slight increase in PDI is most probably due to broadening in size distribution due to coalescence of oil nanodroplets during desorption from the carrier. However, the PDI values were still below 0.7. PDI values bigger than 0.7 indicate that the sample has a broad particle size distribution [67]. Results confirmed the capability of lipid components of SNECT to keep its initial emulsification characteristics after tablet transformation.

#### 3.5.2. In Vitro Dissolution

**Figure 8:**
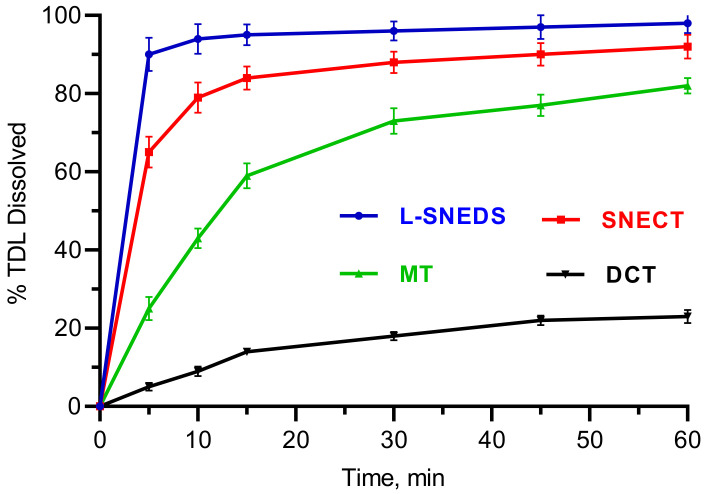
In vitro dissolution profiles tadalafil from liquid self-nanoemulsifying drug delivery systems (L-SNEDDS), self-nanoemulsifying chewable tablets (SNECT), marketed tablets (MT), and direct compressed tablets (DCT).

The in vitro drug dissolution profile of TDL-SNECTS compared to L-SNEDDS, tablets of unprocessed drug (DCT), and marketed tablets (MT) was studied (Figure 8). DCT showed a poor dissolution pattern with a maximum TDL dissolved value of 14% in 15 min. MT, which contains sodium lauryl sulfate acting as a solubilizing agent [68], displayed a drug release up to 59% in the same time. On the other hand, both SNEDDS formulations showed faster drug dissolution profiles (95% and 84% within 15 min for L-SNEDDS and SNECT, respectively). The quicker drug release from SNEDDS formulations is related to the spontaneous formation of nanoemulsions with a large surface area, the molecular dispersed state of drug, as well as wetting and high solubilization capacities of surfactant and cosurfactant mixtures [14,48]. In Figure 8, it was also noticeable that TDL dissolution from SNECT formulation was marginally slower than L-SNEDDS formulation. A plausible explanation is the type of dosage form. Additional steps of tablet disintegration and then desorption of liquid SNEDDS from the excipients surface are required prior the emulsification process. Moreover, excipients such as silicone dioxide still have their impact on the adsorbed SNEDDS, affecting the rate and extent of TDL release [62]. Nonetheless, such a small difference in drug release between L-SNEDDS and SNECT suggests that SNECT retained the self-nanoemulsifying character and the consequent ability to enhance dissolution of TDL.

#### 3.5.3. Stability Study

TDL-SNECT were also tested for stability in various simulated fluids that would be encountered under physiological situations when supplied orally. According to the obtained results, the prepared TDL tablets did not demonstrate any obvious changes in size or PDI (Table 6). The oil phase and the solid carrier offered a protection against instability in simulated GI fluids [47]. SNEDDS are predicted to face varying folds of dilution of GI fluids during their passage through the GI tract. SNECT were found to be intact and stable in all dilutions of simulated GI fluids, with no indication of phase separation or drug precipitation (Table 7). There were negligible variations in the tested parameters before and after three months of accelerated storage at 40 °C and 75 percent relative humidity (RH) (Table 8). This suggests that the formulation was reasonably stable under both storage conditions.

#### 3.5.4. Bioavailability Study

**Figure 9:**
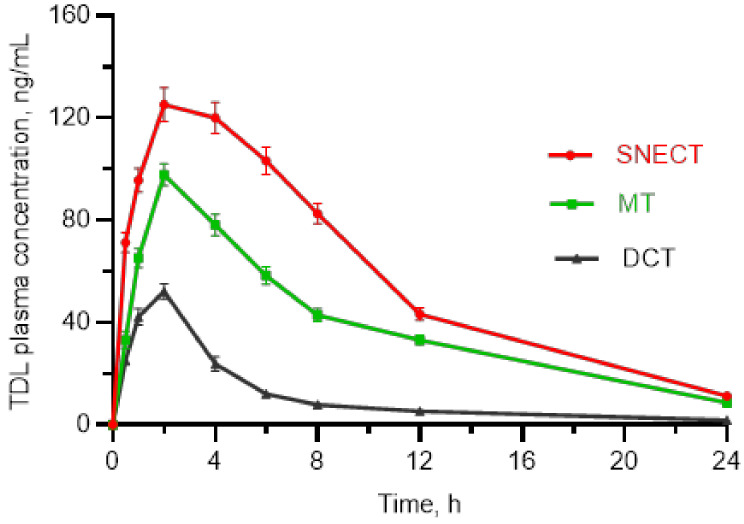
Rat plasma concentrations of tadalafil against time, following oral administration of tested formulations, self-nanoemulsifying chewable tablets (SNECT), marketed tablets (MT), and direct compressed tablets (DCT).

Figure 9 shows the plasma concentration–time profiles of TDL-loaded SNECT and their related DCT and MT. The pharmacokinetic parameters are listed in Table 9. TDL-loaded SNECT displayed a higher absorption profile than DCT at each time point, which could be due to TDL’s low water solubility and poor dissolving capabilities in its unprocessed form. The SNECT formulation generated a mean C_max_ value of 125.2 ± 6.6 ng/mL, which was 2.3-fold higher than the C_max_ obtained by the same dose of TDL-DCT (54.2 ± 3.2 ng/mL, *p* < 0.05). As a marker of bioavailability, AUC_0–24 h_ following the administration of SNECT was 5.33-times higher than after dosing of DCT, i.e., 1403.0 ± 70.2 ng × h/mL and 263.4 ± 14.7 ng × h/mL, respectively (*p* < 0.05). The time of peak plasma concentration (t_max_) observed at 2.0 ± 0.3 h for SNECT was slightly faster as compared to its DCT (*p* > 0.05). The t_1/2_ value of SNECT was also longer than that of DCT: 6.63 ± 0.8 h vs. 4.90 ± 0.4 h. The C_max_ and AUC values of SNECT formulation were also significantly greater than those of MT (*p* < 0.05, Table 9). Hence, the developed TDL-loaded SNECT was efficient to improve TDL bioavailability. The SNECT formulation rapidly generates oily, dispersed nanoemulsions in GI tracts. Here, TDL is presented in a dissolved form with a broad, interfacial surface area for absorption, resulting in increased oral bioavailability. Surface-active compounds may also have further effects, such as enhancing membrane fluidity to aid transcellular absorption and opening tight junctions to allow paracellular transport.

## 4. Conclusions

In the current study, an innovative TDL-SNECT was successfully formulated after L-SNEDDS solidification using a carrier mix of silicon dioxide and MCC. Droplet size remained below 200 nm for both TDL-LSNEDDS and TDL-SNECT. X-ray and DSC investigations revealed that the existence of TDL is in a solubilized state within the solid formulations. Results also revealed the enhancement of rate and extent of TDL dissolution and absorption for SNECT formulation. This would be advantageous for successfully augmenting the oral bioavailability of TDL. Effectively, SNECT administration is advantageous to present TDL in immediate-release formulations with augmented bioavailability. The manufacture of SNECT was a simple procedure that included dissolving the medication in the SNEDD system, solidification, and tableting. The processes and compounds used to create SNECT are inexpensive and uncomplicated. Because of its simplicity of manufacturing and remarkable physical stability, SNECT could be successful for a variety of commercial applications. Further toxicity studies are required to evaluate the safety of the formulation via oral delivery.

## Figures and Tables

**Table 1:** Composition of TDL loaded L-SNEDDS formulations.

Code	Composition		
	Oil ^a^	S ^b^	CoS ^c^
F1	5	4	1
F2	5	6	1.5
F3	5	8	2
F4	5	12	3
F5	5	16	4
F6	5	20	5

^a^ Cinnamon essential oil (CEO); ^b^ Cremophor^®^ RH 40 (CR); ^c^ polyethylene glycol 400 (PEG 400). L-SNEDD, liquid self-nanoemulsifying drug delivery systems.

**Table 2:** Ingredients of tadalafil tablet formulations.

Ingredient (mg/Tablet)	Formulation Codes	
	SNECT	DCT
Tadalafil	2.5	2.5
Cinnamon essential oil	50	-
Cremophor^®^ RH 40	80	-
PEG 400	20	-
MCC	140	140
Silicon dioxide	140	140
Mannitol	250	350
Polyplasdone^TM^ XL 10	10	10
Steviana	5	5
Magnesium stearate	2.5	2.5

SNECT, self-nanoemulsified chewable tablets; DCT, direct compressible tablets; MCC, microcrystalline cellulose.

**Table 3:** Screening of surfactants and cosurfactants for oil emulsification.

Surfactants/Cosurfactants		No. of Inversions	Transmittance %
Surfactants	Tween^®^ 80	23.33 ± 1.42	90.10 ±3.4
	Tween^®^ 20	27.57 ± 2.42	88.22 ± 2.9
	Cremophor^®^ RH 40	13.00 ± 1.42	94.31 ± 1.8
	Labrasol^TM^	36.66 ± 1.42	78.97 ± 4.8
	Span^®^ 80	33.20 ± 1.42	81.70 ± 5.6
Cosurfactants	PEG 400	11.10 ± 1.42	98.50 ± 0.9
	PEG 200	12.80 ± 1.42	96.35 ± 1.3
	PG	29.97± 4.42	83.4 ± 2.5
	Glycerin	33.70 ± 7.42	82.8 ± 2.9

**Table 4:** Characterization of tadalafil-loaded liquid self-nanoemulsifying drug delivery systems (TDL-L-SNEDDS) formulations.

Code	VO	PT (%)	SET (Sec)	Robustness to Dilution	ADS (nm)	PDI	ZP (mV)	Thermodynamic Stability Tests		
								H/C	CT	F/T Test
F1	B	90.1 ± 1.7	39 ± 0.33	No	198 ± 8.7	4.5 ± 1.80	−4.7 ± 0.3	X	-	-
F2	B	92.3 ± 2.4	37 ± 0.43	Yes	222 ± 7.6	3.20 ± 0.97	−5.8 ± 0.27	√	X	-
F3	A	95.7 ± 1.1	29 ± 0.37	Yes	159 ± 4.7	0.24 ± 0.03	−11.9 ± 0.82	√	√	√
F4	A	96.3 ± 1.6	25 ± 0.77	Yes	132 ± 6.8	0.45 ± 0.09	−17.8 ± 0.18	√	√	√
F5	A	97.9 ± 1.3	23 ± 0.34	Yes	45 ± 6.9	1.24 ± 0.16	−18.1 ± 0.25	√	√	√
F6	A	98.8 ± 1.8	20 ± 0.29	Yes	32 ± 4.6	1.35 ± 0.37	−19.7 ± 0.41	√	√	√

Mean ± SD; *n* = 3; SD, standard deviation; L-SNEDD, liquid self-nanoemulsifying drug delivery systems; VO, visual observation; PT, percentage transmittance; SET, self-emulsification time; AGS, average droplet size (ADS); PDI, polydispersity index; ZP, zeta potential; CT, centrifugation test; H/C cycle, heating cooling test; F/T test, freeze/thaw test.

**Table 5:** Flow and reconstitution properties of tadalafil-loaded self-nanoemulsifying granules (TDL-SNEG).

Code	CI	HR	AR	ADS (nm)	(PDI)	ZP (mV)
TDL-SNEGS	12.2 ± 1.1	1.23 ± 0.14	28.3 ± 1.6	165 ± 3.7	0.49 ± 0.1	−8.7 ± 1.1

Mean ± SD; *n* = 3; SD, standard deviation; CI, Carr’s index; HR, Hausner ratio; AR, angle of repose; ADS, average droplet size; PDI, polydispersity index; ZP, zeta potential.

**Table 6:** Stability of tadalafil-loaded self-nanoemulsifying chewable tablets (SNECT) in GI fluids.

	Simulated Gastric Fluid (SGF)		Simulated Intestinal Fluid (SIF)	
	Initial	After 2 h	Initial	After 6 h
ADS	163 ± 7.8	165 ± 8.3	161 ± 4.5	169 ± 9.5
PDI	0.48 ± 0.4	0. 49 ± 0.4	0.48 ± 0.2	0.50 ± 0.03

**Table 7:** Effect of dilution with simulated GI fluids on self-nanoemulsifying chewable tablets (SNECT) characteristics.

Formulation	Dilution Fold	SGF		SIF	
		ADS	PDI	ADS	PDI
TDL-SNECT	10	165 ± 8.3	0.49 ± 0.04	166 ± 7.5	0.50 ± 0.04
	100	169 ± 7.9	0.42 ± 0.03	169 ± 8.6	0.48 ± 0.05
	200	169 ± 6.6	0.41 ± 0.02	174 ± 9.9	0.44 ± 0.03
	400	173 ± 7.1	0.46 ± 0.07	176 ± 7.7	0.49 ± 0.05
	800	174 ± 8.3	0.51 ± 0.05	179 ± 8.3	0.51 ± 0.02

**Table 8:** Results of accelerated stability tests.

Formulation	Storage Conditions	Parameter	Initial	After 3 Months
TDL-SNECT	40 °C ±/ 75% RH	Appearance	Light yellow, round tablets	Light yellow, round tablets
		ADS (nm)	169 ± 8.70	172 ± 6.30
		PDI	0.53 ± 0.05	0.51 ± 0.09
		Drug content (%)	96.42 ± 1.1	95.73 ± 2.6
		Friability (%)	0.29 ± 0.01	0.28 ± 0.01
		Hardness (kg/cm^2^)	4.23 ± 0.33	4.25 ± 0.45
		Disintegration time (Sec)	49 ± 3.7	51 ± 2.9

**Table 9:** Pharmacokinetic parameters of TDL in rats after oral administration (1 mg/kg, n = 6).

PK Parameter	SNECT	DCT	MT
C_max_, ng mL^−1^	125.2 ± 6.6	54.2 ± 3.2	97.8 ± 4.3
t_max_ (h)	2.0 ± 0.3	2.1 ± 0.2	1.90 ± 0.3
AUC _0–24 h_ (ng × h × mL^−1^)	1403.0 ± 70.2	263.4 ± 14.7	930.0 ± 47.4
t _½_ (h)	6.63 ± 0.8	4.90 ± 0.4	6.70 ± 0.3

PK, pharmacokinetic parameter; SNECT, self-nanoemulsified chewable tablets; DCT, direct compressible tablets; MT, marketed tablets.

## Data Availability

Data sharing is not applicable to this article.

## References

[1] Bolat M.S., Akdeniz E., Asci R., Erdemir F., Cinar O., Tomak L. (2017). Ureterorenoscopy with stenting and its effect on male sexual function: A controlled randomised prospective study. Andrologia.

[2] Shamloul R., Ghanem H. (2013). Erectile dysfunction. Lancet.

[3] Dong J.-Y., Zhang Y.-H., Qin L.-Q. (2011). Erectile dysfunction and risk of cardiovascular disease: Meta-analysis of prospective cohort studies. J. Am. Coll. Cardiol..

[4] Velurajah R., Brunckhorst O., Waqar M., McMullen I., Ahmed K. (2022). Erectile dysfunction in patients with anxiety disorders: A systematic review. Int. J. Impot. Res..

[5] Albersen M., Linsen L., Tinel H., Sandner P., Van Renterghem K. (2013). Synergistic effects of bay 60-4552 and vardenafil on relaxation of corpus cavernosum tissue of patients with erectile dysfunction and clinical phosphodiesterase type 5 inhibitor failure. J. Sex. Med..

[6] Bella A.J., Brock G.B. (2003). Tadalafil in the treatment of erectile dysfunction. Curr. Urol. Rep..

[7] Baek J.-S., Cho C.-W. (2016). Transdermal delivery of tadalafil using a novel formulation. Drug Deliv..

[8] Carson C., Shabsigh R., Segal S., Murphy A., Fredlund P. (2005). Efficacy, safety, and treatment satisfaction of tadalafil versus placebo in patients with erectile dysfunction evaluated at tertiary-care academic centers. Urology.

[9] Coward R.M., Carson C.C. (2008). Tadalafil in the treatment of erectile dysfunction. Ther. Clin. Risk Manag..

[10] Mobarak D., Salah S., Ghorab M. (2019). Improvement of dissolution of a class ii poorly water-soluble drug, by developing a five-component self-nanoemulsifying drug delivery system. J. Drug Deliv. Sci. Technol..

[11] Badr-Eldin S.M., Elkheshen S.A., Ghorab M.M. (2017). Improving tadalafil dissolution via surfactant-enriched tablets approach: Statistical optimization, characterization, and pharmacokinetic assessment. J. Drug Deliv. Sci. Technol..

[12] Fitria A., Hanifah S., Chabib L., Uno A.M., Munawwarah H., Atsil N., Pohara H.A., Weuanggi D.A., Syukri Y. (2021). Design and characterization of propolis extract loaded self-nano emulsifying drug delivery system as immunostimulant. SJP.

[13] Buya A.B., Beloqui A., Memvanga P.B., Préat V. (2020). Self-nano-emulsifying drug-delivery systems: From the development to the current applications and challenges in oral drug delivery. Pharmaceutics.

[14] Dalal L., Allaf A.W., El-Zein H. (2021). Formulation and in vitro evaluation of self-nanoemulsifying liquisolid tablets of furosemide. Sci. Rep..

[15] Gawin-Mikołajewicz A., Nartowski K.P., Dyba A.J., Gołkowska A.M., Malec K., Karolewicz B. (2021). Ophthalmic nanoemulsions: From composition to technological processes and quality control. Mol. Pharm..

[16] Dash R.N., Mohammed H., Humaira T., Ramesh D. (2015). Design, optimization and evaluation of glipizide solid self-nanoemulsifying drug delivery for enhanced solubility and dissolution. SJP.

[17] Alwadei M., Kazi M., Alanazi F.K. (2019). Novel oral dosage regimen based on self-nanoemulsifying drug delivery systems for codelivery of phytochemicals–curcumin and thymoquinone. SJP.

[18] Kim D.H., Kim J.Y., Kim R.M., Maharjan P., Ji Y.-G., Jang D.-J., Min K.A., Koo T.-S., Cho K.H. (2018). Orlistat-loaded solid snedds for the enhanced solubility, dissolution, and in vivo performance. Int. J. Nanomed..

[19] Parmar K., Patel J., Sheth N. (2015). Self nano-emulsifying drug delivery system for embelin: Design, characterization and in-vitro studies. Asian J. Pharm. Sci..

[20] Wang L., Dong J., Chen J., Eastoe J., Li X. (2009). Design and optimization of a new self-nanoemulsifying drug delivery system. J. Colloid Interface Sci..

[21] Subramanian P. (2021). Lipid-based nanocarrier system for the effective delivery of nutraceuticals. Molecules.

[22] Soliman K.A., Ibrahim H.K., Ghorab M.M. (2016). Formulation of avanafil in a solid self-nanoemulsifying drug delivery system for enhanced oral delivery. Eur. J. Pharm. Sci..

[23] Krstić M., Ražić S., Djekic L., Dobričić V., Momčilović M., Vasiljevic D., Ibrić S. (2015). Application of a mixture experimental design in the optimization of the formulation of solid self-emulsifying drug delivery systems containing carbamazepine. Lat. Am. J. Pharm..

[24] Beg S., Katare O.P., Saini S., Garg B., Khurana R.K., Singh B. (2016). Solid self-nanoemulsifying systems of olmesartan medoxomil: Formulation development, micromeritic characterization, in vitro and in vivo evaluation. Powder Technol..

[25] Vohra A.M., Patel C.V., Kumar P., Thakkar H.P. (2017). Development of dual drug loaded solid self microemulsifying drug delivery system: Exploring interfacial interactions using qbd coupled risk based approach. J. Mol. Liq..

[26] Kang J.H., Oh D.H., Oh Y.-K., Yong C.S., Choi H.-G. (2012). Effects of solid carriers on the crystalline properties, dissolution and bioavailability of flurbiprofen in solid self-nanoemulsifying drug delivery system (solid snedds). Eur. J. Pharm. Biopharm..

[27] Tang B., Cheng G., Gu J.-C., Xu C.-H. (2008). Development of solid self-emulsifying drug delivery systems: Preparation techniques and dosage forms. Drug Discov. Today.

[28] Nanda Kishore R., Yalavarthi P.R., Vadlamudi H.C., Vandana K.R., Rasheed A., Sushma M. (2015). Solid self microemulsification of atorvastatin using hydrophilic carriers: A design. Drug Dev. Ind. Pharm..

[29] Krstic M., Djuris J., Petrovic O., Lazarevic N., Cvijic S., Ibric S. (2017). Application of the melt granulation technique in development of lipid matrix tablets with immediate release of carbamazepine. J. Drug Deliv. Sci. Technol..

[30] Tarate B., Chavan R., Bansal K.A. (2014). Oral solid self-emulsifying formulations: A patent review. Recent Pat. Drug Deliv. Formul..

[31] Beg S., Jena S.S., Patra C.N., Rizwan M., Swain S., Sruti J., Rao M.E.B., Singh B. (2013). Development of solid self-nanoemulsifying granules (ssnegs) of ondansetron hydrochloride with enhanced bioavailability potential. Colloids Surf. B Biointerfaces.

[32] Wang Z., Sun J., Wang Y., Liu X., Liu Y., Fu Q., Meng P., He Z. (2010). Solid self-emulsifying nitrendipine pellets: Preparation and in vitro/in vivo evaluation. Int. J. Pharm..

[33] El-Badry M., Haq N., Fetih G., Shakeel F. (2014). Solubility and dissolution enhancement of tadalafil using self-nanoemulsifying drug delivery system. J. Oleo Sci..

[34] Sonawane P.H., Panzade P.S., Kale M.A. (2013). Rapid estimation of tadalafil by reverse-phase high-performance liquid chromatography method in bulk and tablet formulation. Indian J. Pharm. Sci..

[35] Ahmed S.A., Alalawi A.M., Shehata A.M., Alqurshi A.A., Alahmadi Y.M., Ali H.S. (2022). Fabric phase sorptive extraction coupled with uplc-esi-ms/ms method for fast and sensitive quantitation of tadalafil in a bioequivalence study. SPJ.

[36] Abd-Elhakeem E., Teaima M.H.M., Abdelbary G.A., El Mahrouk G.M. (2019). Bioavailability enhanced clopidogrel -loaded solid snedds: Development and in-vitro/in-vivo characterization. J. Drug Deliv. Sci. Technol..

[37] Choudhury H., Gorain B., Karmakar S., Biswas E., Dey G., Barik R., Mandal M., Pal T.K. (2014). Improvement of cellular uptake, in vitro antitumor activity and sustained release profile with increased bioavailability from a nanoemulsion platform. Int. J. Pharm..

[38] Yen C.-C., Chang C.-W., Hsu M.-C., Wu Y.-T. (2017). Self-nanoemulsifying drug delivery system for resveratrol: Enhanced oral bioavailability and reduced physical fatigue in rats. Int. J. Mol. Sci..

[39] Patel G., Shelat P., Lalwani A. (2016). Statistical modeling, optimization and characterization of solid self-nanoemulsifying drug delivery system of lopinavir using design of experiment. Drug Deliv..

[40] Nasr A., Gardouh A., Ghorab M. (2016). Novel solid self-nanoemulsifying drug delivery system (s-snedds) for oral delivery of olmesartan medoxomil: Design, formulation, pharmacokinetic and bioavailability evaluation. Pharmaceutics.

[41] Nekkanti V., Karatgi P., Prabhu R., Pillai R. (2010). Solid self-microemulsifying formulation for candesartan cilexetil. AAPS PharmSciTech.

[42] Yadav P., Rastogi V., Verma A. (2020). Application of box–behnken design and desirability function in the development and optimization of self-nanoemulsifying drug delivery system for enhanced dissolution of ezetimibe. Future J. Pharm. Sci..

[43] Goh P.S., Ng M.H., Choo Y.M., Amru N.B., Chuah C.H. (2015). Production of nanoemulsions from palm-based tocotrienol rich fraction by microfluidization. Molecules.

[44] Nair A.B., Singh B., Shah J., Jacob S., Aldhubiab B., Sreeharsha N., Morsy M.A., Venugopala K.N., Attimarad M., Shinu P. (2022). Formulation and evaluation of self-nanoemulsifying drug delivery system derived tablet containing sertraline. Pharmaceutics.

[45] Mehanna M.M., Mneimneh A.T., Domiati S., Allam A.N. (2020). Tadalafil-loaded limonene-based orodispersible tablets: Formulation, in vitro characterization and in vivo appraisal of gastroprotective activity. Int. J. Nanomed..

[46] Mahmoud E.A., Bendas E.R., Mohamed M.I. (2009). Preparation and evaluation of self-nanoemulsifying tablets of carvedilol. AAPS PharmSciTech.

[47] Jain S., Dongare K., Nallamothu B., Parkash Dora C., Kushwah V., Katiyar S.S., Sharma R. (2022). Enhanced stability and oral bioavailability of erlotinib by solid self nano emulsifying drug delivery systems. Int. J. Pharm..

[48] Sawatdee S., Atipairin A., Sae Yoon A., Srichana T., Changsan N., Suwandecha T. (2019). Formulation development of albendazole-loaded self-microemulsifying chewable tablets to enhance dissolution and bioavailability. Pharmaceutics.

[49] Lee J.H., Oh J.-H., Lee Y.-J. (2013). Simple and sensitive liquid chromatography–tandem mass spectrometry methods for quantification of tadalafil in rat plasma: Application to pharmacokinetic study in rats. Arch. Pharm. Res..

[50] Balakumar K., Raghavan C.V., Selvan N.T., Prasad R.H., Abdu S. (2013). Self nanoemulsifying drug delivery system (snedds) of rosuvastatin calcium: Design, formulation, bioavailability and pharmacokinetic evaluation. Colloids Surf. B Biointerfaces.

[51] Ali H.H., Hussein A.A. (2017). Oral nanoemulsions of candesartan cilexetil: Formulation, characterization and in vitro drug release studies. AAPS Open.

[52] Akhtar J., Siddiqui H.H., Fareed S., Badruddeen K.M., Aqil M. (2016). Nanoemulsion: For improved oral delivery of repaglinide. Drug Deliv..

[53] Onder A., Yilmaz-Oral D., Jerkovic I., Akdemir A.O., Gur S. (2019). Evaluation of relaxant responses properties of cinnamon essential oil and its major component, cinnamaldehyde on human and rat corpus cavernosum. Int. Braz. J. Urol..

[54] Patel J., Dhingani A., Tilala J., Raval M., Sheth N. (2014). Formulation and development of self-nanoemulsifying granules of olmesartan medoxomil for bioavailability enhancement. Part. Sci. Technol..

[55] Azeem A., Rizwan M., Ahmad F.J., Iqbal Z., Khar R.K., Aqil M., Talegaonkar S. (2009). Nanoemulsion components screening and selection: A technical note. AAPS PharmSciTech.

[56] Wei Y., Ye X., Shang X., Peng X., Bao Q., Liu M., Guo M., Li F. (2012). Enhanced oral bioavailability of silybin by a supersaturatable self-emulsifying drug delivery system (s-sedds). Colloids Surf. A Physicochem. Eng..

[57] Dixit R.P., Nagarsenker M.S. (2008). Self-nanoemulsifying granules of ezetimibe: Design, optimization and evaluation. Eur. J. Pharm. Sci..

[58] Ke Z., Hou X., Jia X.-B. (2016). Design and optimization of self-nanoemulsifying drug delivery systems for improved bioavailability of cyclovirobuxine d. Drug Des. Devel. Ther..

[59] Li P., Ghosh A., Wagner R.F., Krill S., Joshi Y.M., Serajuddin A.T.M. (2005). Effect of combined use of nonionic surfactant on formation of oil-in-water microemulsions. Int. J. Pharm..

[60] Anton N., Vandamme T.F. (2011). Nano-emulsions and micro-emulsions: Clarifications of the critical differences. Pharm. Res..

[61] Rehman F.U., Shah K.U., Shah S.U., Khan I.U., Khan G.M., Khan A. (2017). From nanoemulsions to self-nanoemulsions, with recent advances in self-nanoemulsifying drug delivery systems (snedds). Expert Opin. Drug Deliv..

[62] Weerapol Y., Limmatvapirat S., Nunthanid J., Sriamornsak P. (2014). Self-nanoemulsifying drug delivery system of nifedipine: Impact of hydrophilic-lipophilic balance and molecular structure of mixed surfactants. AAPS PharmSciTech.

[63] Muqtader Ahmed M., Fatima F., Abul Kalam M., Alshamsan A., Soliman G.A., Shaikh A.A., Alshahrani S.M., Aldawsari M.F., Bhatia S., Khalid Anwer M. (2020). Development of spray-dried amorphous solid dispersions of tadalafil using glycyrrhizin for enhanced dissolution and aphrodisiac activity in male rats. SPJ.

[64] Ohwoavworhua F.O., Adelakun T.A., Okhamafe A.O. (2009). Processing pharmaceutical grade microcrystalline cellulose from groundnut husk: Extraction methods and characterization. Int. J. Green Pharm..

[65] Wei Y., Ling Y., Su M., Qin L., Zhang J., Gao Y., Qian S. (2018). Characterization and stability of amorphous tadalafil and four crystalline polymorphs. Chem. Pharm. Bull..

[66] Fissan H., Ristig S., Kaminski H., Asbach C., Epple M. (2014). Comparison of different characterization methods for nanoparticle dispersions before and after aerosolization. Anal. Methods.

[67] Danaei M., Dehghankhold M., Ataei S., Hasanzadeh Davarani F., Javanmard R., Dokhani A., Khorasani S., Mozafari M.R. (2018). Impact of particle size and polydispersity index on the clinical applications of lipidic nanocarrier systems. Pharmaceutics.

[68] Wlodarski K., Tajber L., Sawicki W. (2016). Physicochemical properties of direct compression tablets with spray dried and ball milled solid dispersions of tadalafil in pvp-va. Eur. J. Pharm. Biopharm..

